# Consecutive Controlled Case Series on Effectiveness of Opipramol in Severe Sleep Bruxism Management—Preliminary Study on New Therapeutic Path

**DOI:** 10.3390/brainsci11020146

**Published:** 2021-01-22

**Authors:** Mieszko Wieckiewicz, Helena Martynowicz, Tomasz Wieczorek, Anna Wojakowska, Katarzyna Sluzalec-Wieckiewicz, Pawel Gac, Rafal Poreba, Grzegorz Mazur, Efraim Winocur, Joanna Smardz

**Affiliations:** 1 Department of Experimental Dentistry, Wroclaw Medical University, 50-425 Wroclaw, Poland; joannasmardz1@gmail.com; 2 Department and Clinic of Internal Medicine, Occupational Diseases, Hypertension and Clinical Oncology, Wroclaw Medical University, 50-556 Wroclaw, Poland; helenamar@poczta.onet.pl (H.M.); ania.wojakowska@wp.pl (A.W.); sogood@poczta.onet.pl (R.P.); grzegorzmaz@yahoo.com (G.M.); 3 Department and Clinic of Psychiatry, Wroclaw Medical University, 50-367 Wroclaw, Poland; dobrewieczorki@gmail.com; 4 Private Dental Practice, 53-407 Wroclaw, Poland; karas60@o2.pl; 5 Department of Hygiene, Wroclaw Medical University, 50-345 Wroclaw, Poland; pawelgac@interia.pl; 6 Department of Oral Rehabilitation, The Maurice and Gabriela Goldschleger School of Dental Medicine, Tel Aviv University, Tel Aviv 6139001, Israel; winocur@tauex.tau.ac.il

**Keywords:** sleep bruxism, polysomnography, opipramol, tricyclic antidepressants

## Abstract

Background: Sleep bruxism (SB) management aims to reduce the number and magnitude of bruxism episodes per hour of a patient’s sleep and, therefore, reduce the potentially negative clinical consequences. Opipramol belongs to the group of tricyclic antidepressants (TCAs) and is considered as an atypical TCA, as it acts primarily as a sigma receptor agonist. This study aimed to preliminarily determine the effectiveness of opipramol in the management of severe SB. Methods: A total of 19 otherwise healthy participants with severe SB diagnosed during stage I video polysomnography (vPSG) were subjected to an 8-week pharmacotherapy trial with a 100 mg bedtime daily dose of opipramol and were then analyzed by control stage II vPSG. Results: The participants included 14 females and 5 males, aged 20–47 years (mean ± standard deviation: 32.32 ± 8.12). A comparison of stage I and II vPSG recordings showed a decrease in all the studied SB parameters in 78.85% of participants. Only in a small group of participants (15.53%) was a non-significant increase of SB parameters observed. Conclusions: A single 100 mg dose of opipramol at bedtime seems to positively affect the reduction of SB in otherwise healthy individuals diagnosed with severe SB. However, the subject requires further research on a larger population including a control group.

## 1. Introduction

In accordance with the 2018 International Consensus, sleep bruxism (SB) and awake bruxism are considered as two separate phenomena [1]. Currently, SB is defined as a masticatory muscle activity that can be either phasic (rhythmic) or tonic (non-rhythmic) and is not a movement disorder or a sleep disorder in otherwise healthy individuals [1]. It is recommended that bruxism can be classified as a behavior, a protective factor, or a risk factor [1,2]. However, in some individuals, for example, those with obstructive sleep apnea (OSA), epilepsy, rapid eye movement (REM) sleep behavior disorder, or gastroesophageal reflux disease, SB can be considered as a sign of a disorder [1,2,3,4,5]. Furthermore, as a masticatory muscle activity, SB can lead to numerous negative clinical consequences, including damage to the dental hard tissues such as tooth wear or cracked teeth, damage to the oral mucosa, prosthodontic complications, fatigue and pain in masticatory muscles, and headache [1,2,3,4,5].

SB is a common behavior, and its prevalence is estimated in 13% of the general population [3]. Due to the occurrence in sleep, the diagnosis of SB is quite challenging [5]. Most commonly, patients report pain in masticatory muscles or tooth wear [1,2,3,4,5]. Possible bruxism can be diagnosed on the basis of positive self-reporting, while probable bruxism can be diagnosed from a positive clinical examination (with or without positive self-reporting). On the other hand, definite bruxism can only be diagnosed using instrumental diagnostic methods. Video polysomnography (vPSG) seems to be the best among the methods available for SB diagnosis [1]. vPSG allows for the assessment of SB both in terms of quantity (bruxism episodes and burst indexes) and qualitative (number of phasic, tonic and mixed episodes). SB is believed to have a multifactorial and complex origin, and to be caused by biological, psychological, and exogenous factors. In addition, genetic factors seem to play a crucial role in its occurrence, but the neurophysiology of SB is still unclear [1,2,3,4,5].

Due to the fact that SB is not considered a disorder, management should be implemented only when patients experience the negative consequences. SB management aims to reduce the number and magnitude of bruxism episodes per hour of a patient’s sleep and, therefore, reduce the risk of occurrence of the negative clinical consequences [4]. Due to the complexity and multifactorial origin of SB and the difficulty in its diagnosis, proper management often requires multi-specialist collaboration and is, therefore, a major clinical challenge [1,2,3,4,5]. Until now, SB has been managed only according to the symptoms using oral appliances [6,7,8,9] and physical therapy [10,11,12]. Among the methods of causal management, sleep hygiene [13,14], botulinum toxin injections [15,16], and pharmacotherapy [17,18] are often described. Several therapeutic agents, such as benzodiazepines, anticonvulsants, beta-blockers, dopamine agents, antidepressants, and muscle relaxants have been tested for the management of SB [18]; in particular, the use of amitriptyline, bromocriptine, clonidine, propranolol, tryptophan, levodopa, and pramipexole were analyzed [17,19]. However, none of these drugs have been introduced to the standard targeted SB management.

Opipramol is a dibenzazepine derivative developed in the 1960s. It belongs to the group of tricyclic antidepressants (TCAs) [20,21,22]. TCAs are currently used for treating a number of disorders such as mood disorders, anxiety disorders, eating disorders, personality disorders, Parkinson’s disease, chronic pain, and insomnia [23,24]. Although opipramol belongs to this group of drugs, it is atypical because most of the TCAs act as monoamine reuptake inhibitors, but opipramol acts primarily as a strong sigma 1 and 2 receptor agonist. It acts also as moderate dopamine D2, serotonin 5-HT2 and histamine H1 receptors antagonist and shows low anticholinergic activity. Opipramol has an anxiolytic, sedative and mood-improving effect, while it has less antidepressant properties [20,21,22]. When applied in the evening, it has sleep-improving effect [25,26]. Gerlach et al. reported that evening opipramol significantly improved sleep quality, and in particular the frequency of awakening at night was reduced. Although the minimal recommended dosage starts at 50 mg, these effects could be observed predominantly after 100 mg opipramol [25]. Side effects of low-dose opipramol intake are rather rare [20,21,22]. Although other TCAs such as amitriptyline [17] have been investigated for their effectiveness in reducing bruxism episodes, no studies have examined the effectiveness of opipramol in this issue. As SB is often reported to be associated with awakening [1,2], opipramol seems to perfectly meet the criteria of a potentially effective and multifaceted drug.

The high prevalence and complexity of SB and the multitude of its clinical manifestations have increased the need to search for an effective drug that would reduce the number of bruxism episodes, while not adversely affecting the sleep parameters and the overall well-being of the patients. Therefore, the purpose of this study was to preliminarily determine the effectiveness of low-dose opipramol in the management of severe SB. It has been hypothesized that opipramol may be a possible effective drug in reducing severe SB.

## 2. Materials and Methods

This prospective consecutive controlled case series was prepared in accordance to the preferred reporting of case series in surgery (PROCESS) guidelines (https://www.processguideline.com/).

### 2.1. Participants

The participants of this prospective consecutive controlled case series were adult patients of the Outpatient Clinic for Temporomandibular Disorders operating at the Department of Experimental Dentistry of the Wroclaw Medical University in Poland. The study was conducted from October 2017 until December 2019. All the participants provided written informed consent before inclusion. The study was approved by the Ethical Committee of the Wroclaw Medical University (ID KB-195/2017) and conducted in accordance with the Declaration of Helsinki. The information regarding clinical trial registration is available at www.ClinicalTrials.gov (identifier NCT03083405).

### 2.2. Recruitment

The patients attending the Outpatient Clinic for Temporomandibular Disorders were subjected to a thorough medical interview, and an intra- and extraoral clinical examination based on the Diagnostic Criteria for Temporomandibular Disorders focusing on self-reporting (including bed partner reporting) and the signs and symptoms of bruxism. The procedures were performed by experienced dentists. Patients diagnosed with probable SB in accordance with the Third Edition of the International Classification of Sleep Disorders (ICSD-3) published by the American Academy of Sleep Medicine (AASM) [5] were subjected to single-night vPSG. None of the included patients used oral devices or get any other treatment which could potentially affect intensity of sleep bruxism. Patients diagnosed with severe definitive SB after the stage I vPSG (the number of bruxism episodes per hour of sleep—bruxism episode index (BEI) > 4) were included in the study. The patients were recruited from October 2017 to December 2019. No psychiatric evaluation was carried out.

### 2.3. Inclusion Criteria

The general inclusion criteria were as follows: age ≥ 18, willingness to participate in the study, and diagnosis of definitive severe SB confirmed in stage I vPSG.

### 2.4. Exclusion Criteria

The general exclusion criteria were as follows: presence of neurological disorders and/or neuropathic pain, active inflammation, OSA, active malignancy, severe mental disorders, or cognitive disability; treatment with or addiction to any analgesic and/or drugs affecting muscle functions and breathing; history of pharyngeal surgery for the treatment of OSA; and contraindications for opipramol pharmacotherapy.

### 2.5. Stage I Video Polysomnography (vPSG)

Patients diagnosed with probable SB in accordance to clinical examination underwent a single-night vPSG with NoxA1 (Nox Medical, Reykjavík, Iceland) in the Sleep Laboratory operating at the Department and Clinic of Internal Medicine, Occupational Diseases, Hypertension and Clinical Oncology of the Wroclaw Medical University. Recordings were carried out between 10.00 p.m. and 6.00 a.m., taking into account the preferences and sleeping habits of the patients. The electrodes were arranged in a standard manner including international 10–20 system and following the manufacturer’s recommendations. The only modification relative to the standard distribution of electrodes was the placement of bipolar leads for electromyographic recording from both sides of the origin and insertion of masseter muscles to allow recognizing and recording the SB episodes.

The following standard elements of vPSG were examined: electroencephalographic, electrocardiographic, electrooculographic, and electromyographic recordings from the chin area and bilaterally from the masseter muscles; recording of abdominal and thoracic breathing activity; body position; and audio as well as video recording. To record the level of saturation, pulse, and plethysmographic data, a NONIN WristOx2 3150 pulse oximeter (Nonin Medical Inc., Plymouth, MN, USA) was used. Use of Noxturnal software (Nox Medical, Reykjavík, Iceland) allowed restoring the full vPSG record. A qualified and experienced physician scored and analyzed the vPSG recordings adhering to the AASM guidelines [27].

Polysomnograms were assessed in 30-s epochs in accordance with the 2013 AASM standard criteria for sleep scoring [27].

#### 2.5.1. Sleep Bruxism (SB) Parameters

SB was assessed using bilateral masseter electromyography (EMG) and by evaluation of the audio–video recordings. The following indexes were included in the assessment: BEI, bruxism burst index (BBI), phasic bruxism (more than three cyclic phasic EMG increases lasting 0.25–2 s), tonic bruxism (episodes lasting >2 s), mixed bruxism, apnea to bruxism, and arousal to bruxism. The new SB episodes were scored after at least 3 s of stable electromyography and when the activity was at least twice the amplitude of the background electromyography [5,27]. On the basis of BEI (number of bruxism episodes per hour of sleep), SB was qualified as irrelevant (BEI < 2), mild to moderate (BEI 2–4), or severe (BEI > 4) [5].

#### 2.5.2. Respiratory Event Parameters

Abnormal respiratory events were scored by evaluating the pressure airflow signal in accordance with the standard criteria of the AASM Task Force [27]. Apnea was defined as the absence of airflow for ≥10 s, while hypopnea was defined as a reduction in the breathing amplitude by ≥30% for ≥10 s accompanied by a ≥3% decline in blood oxygen saturation or arousal [5]. The following indexes were included in the assessment: apnea/hypopnea index (AHI); oxygen desaturation index (ODI); snore index; average, minimal, and maximal SpO_2_; SpO_2_ < 90%; and average desaturation drop.

#### 2.5.3. Sleep Parameters

Sleep parameters were assessed using the 2013 AASM standard criteria for sleep scoring. The following indexes were included in the assessment: total sleep time (TST), sleep latency (SL), rapid eye movement (REM) latency (REML), wake after sleep onset (WASO), sleep efficiency (SE), non-REM 1 sleep stage (N1), non-REM 2 sleep stage (N2), non-REM 3 sleep stage (N3), and arousals.

### 2.6. Opipramol Pharmacotherapy

Based on the results of stage I vPSG, otherwise healthy patients diagnosed with severe SB (BEI > 4) and having no contraindications were qualified to receive oral opipramol treatment. The patients were prescribed to take a single daily dose of 100 mg opipramol dihydrochloride (Pramolan, Polpharma SA, Poland) at bedtime for the next 8 weeks. We decided on the use of a 100 mg dose because it was previously reported as having a sleep-improving effect [25]. After 4 weeks, the patients underwent a clinical reevaluation to observe for possible side effects of pharmacotherapy and were referred to stage II vPSG.

### 2.7. Stage II vPSG

Stage II vPSG was performed as a control, after 8 weeks of pharmacotherapy, in the same manner and assessing the same parameters as stage I vPSG.

### 2.8. Statistical Methods

Statistical analysis focusing on a comparison of stage I and II vPSG parameters using paired data was performed using the Statistica version 13 software (Dell Inc., Round Rock, TX, USA). A Shapiro–Wilk test was performed to test the normal distribution of the data. Student’s *t*-test for parametric data and the Mann–Whitney U-test for nonparametric data were carried out to examine the significance of differences in the mean values between the study stages. A correlation analysis was performed to determine the relationship between the studied variables. Statistical significance was set at *p* < 0.05 in all the analyses.

## 3. Results

Thirty otherwise healthy adult participants diagnosed with severe SB during stage I vPSG (BEI > 4) were included in the study. They were subjected to a clinical reevaluation after 4 weeks of the opipramol pharmacotherapy to observe for side effects caused by the drug intake. None of them reported any severe side effects, while 5 reported transient sleepiness within the first few days of opipramol oral administration. All the participants were directed to stage II vPSG, but only 19 completed the examination and, therefore, constituted the study group. The remaining 11 participants did not report to stage II vPSG possibly due to a lack of further willingness to participate in the study. These patients were not taken into consideration in the analysis and presentation of the results.

The study group included 14 females and 5 males, aged 20–47 years (mean ± standard deviation: 32.32 ± 8.12).

The descriptive statistics of all the studied stage I and stage II vPSG parameters are presented in Table 1; Table 2, respectively. Table 3 shows the changes in these parameters after pharmacotherapy.

### 3.1. Influence of Opipramol on SB

The participants were classified into three groups in which (1) SB decreased, (2) SB remained on the same level and (3) SB increased. When considering the decrease of SB also a level difference was reported: from severe (BEI > 4) to mild-moderate (BEI 2–4) and from severe to irrelevant (BEI < 2).

A decrease of BEI was reported in 15 (78.95%) participants (group 1), while in 2 (15.53%) participants BEI remained at almost the same level (group 2) and an increase was reported by 2 (15.53%) participants (group 3). An improvement of BEI from severe to irrelevant was reported by 4 (21.05%) participants and from severe to mild–moderate by 5 (26.32%) participants, while in 6 (31.58%) participants BEI remained at the severe level.

The analysis of SB parameters performed between stage I and stage II vPSGs showed a decrease in the mean value of all the studied SB parameters in all the participants (Table 3). Furthermore, a comparison of SB parameters before and after opipramol treatment showed a statistically significant decrease in BEI (*p* = 0.00), phasic bruxism (*p* = 0.00), tonic bruxism (*p* = 0.03), and arousal to bruxism (*p* = 0.04) after pharmacotherapy. The BEI decrease after pharmacotherapy was statistically significantly higher in those who had a higher initial BEI. Participants with a BEI of ≥6.6 presented a statistically significantly higher decrease in the index and phasic bruxism after the pharmacotherapy than others (*p* = 0.01 for both comparisons).

The analyses on participant groups showing a decrease (group 1), no change (group 2), and an increase (group 3) in BEI after pharmacotherapy revealed an average decrease of 3.35 in the BEI score in group 1 and an average increase of 2.80 in group 3. The detailed comparative analysis of groups 1 and 3 showed statistically significant differences between them in BEI, BBI, and phasic and mixed bruxism. A statistically significantly higher BEI and a decrease of phasic bruxism (*p* = 0.00 and *p* = 0.00, respectively) were observed in group 1, while a statistically significantly higher BBI and an increase of mixed bruxism were observed in group 3 (Table 4).

### 3.2. Influence of Opipramol on Other Sleep Parameters

No statistically significant differences were found in the following: AHI; ODI; snore; TST; SL; REML; WASO; SE; N1, N2, and N2; REM; arousals; average, minimal, and maximal SpO_2_; SpO_2_ <90%; average desaturation drop; and average and minimal pulse (*p* > 0.05 for all comparisons). Maximal pulse was found to be statistically significantly lower after the pharmacotherapy (*p* = 0.02) (Table 1, Table 2 and Table 3).

### 3.3. Influence of Age and Gender on Studied Parameters during Opipramol Treatment

As mentioned before the study, participants were divided into three groups presenting SB decrease (group 1), SB no change (group 2) and SB increase (group 3). Group 1 consisted of 15 participants (aged 20–47; 11 females and 4 males). Group 2 consisted of 2 participants (2 females aged 34 and 24). Group 3 consisted of 2 participants (1 female aged 30 and 1 male aged 28).

The opipramol pharmacotherapy was associated with statistically significant differences between the genders in AHI, ODI, and N3. The studied males presented a statistically significant increase in AHI and ODI (*p* = 0.02 and *p* = 0.04, respectively) and a decrease in N3 after pharmacotherapy compared to females (Table 5).

However, the increase in AHI and ODI had a subclinical meaning in the male participants. On the other hand, the age of the participants seemed to significantly influence the decrease of BBI and maximal pulse during pharmacotherapy. In the case of older participants, the decrease of BBI was higher, while the decrease of maximal pulse was lower (*p* < 0.05 for both comparisons).

## 4. Discussion

This study is the first to assess the effectiveness of opipramol in the management of severe SB and this medication has not been previously studied for this condition. Due to this fact, this section begins with a comparison of the effectiveness of opipramol with other medications previously studied for the reduction of SB.

Opipramol is a dibenzazepine derivative. It is primarily an anxiolytic and antidepressant and belongs to the group of TCAs [20,21,22]. TCAs are currently used for treating a number of disorders including mood disorders, anxiety disorders, eating disorders, personality disorders, Parkinson’s disease, chronic pain, and insomnia [23,24]. Their main mechanism of action is blocking the reuptake of serotonin and norepinephrine [23]. In addition, they exert an anticholinergic effect and often act as blockers of dopamine and histamine receptors [23]. The effectiveness of TCAs, such as amitriptyline, was also assessed in the reduction of bruxism [17,28,29,30,31]. The mechanism of action of amitriptyline is the inhibition of norepinephrine and serotonin reuptake, where it exerts a balanced effect. It also exhibits a strong anticholinergic effect both in the central and peripheral nervous systems [28,29,30,31]. In a study performed in a group of 10 participants, in which electromyography was recorded for 7 consecutive nights during amitriptyline intake, Mohamed et al. reported that no statistically significant differences were found between amitriptyline-administrated (low dose of 25 mg) and control (placebo) groups in the masticatory muscle activity [28]. Raigrodski et al. also reported similar findings in their study [29]. The effectiveness of amitriptyline in reducing SB was reported only in a study focusing on duloxetine-induced SB in fibromyalgia treatment, but the study did not involve any instrumental analysis of masticatory muscle activity [29]. Moreover, other medications (clonidine [32], bromocriptine [33], propranolol [34], tryptophan [35], levodopa [36,37], and pramipexole [19]) were not reported to be effective in reducing the masticatory muscle activity.

It is worth emphasizing that although opipramol belongs to the TCAs group, in some way it is atypical because most of the TCAs act as monoamine reuptake inhibitors, while opipramol acts primarily as sigma receptor agonist and histamine receptors antagonist. [20,21,22]. It is possible that this unique mechanism of action of opipramol might contribute to its effectiveness in reducing SB.

The results of this study indicate the effectiveness of opipramol in reducing all the parameters associated with SB, including BEI, BBI, phasic bruxism, tonic bruxism, mixed bruxism, apnea to bruxism, and arousal to bruxism. In the major group of participants presenting SB reduction (78.95%), the mean decrease was 3.35 for BEI and 2.85 for BBI. In the minor group of participants presenting SB aggravation (15.53%) the mean increase was 2.8 for BEI and 7.5 for BBI. As the risk of SB serious clinical consequences increases with the number of episodes, the recommended management of this phenomenon focuses on reducing BEI [1]. For 78.95% of the participants of this study BEI was significantly reduced after therapy, which directly translates into a decrease in the risk of SB negative clinical consequences development, even if SB was not completely eliminated.

In addition, opipramol did not influence other studied sleep parameters or even decreased the maximal pulse. It should also be emphasized that none of the study participants reported any severe side effects during the opipramol pharmacotherapy. Rarely reported transient sleepiness can be the effect of opipramol’s histamine receptors blocking action. However, given the reported sleep-improving effects of opipramol [25,26], the sleep quality of participants could even improve after longer use or the effect was not visible because of the small study sample.

Although the exact mechanism of action is not yet clear, this study’s results gave rise to the hypothesis that the effectiveness of opipramol in SB could be associated with the fact that it is a sigma receptor agonist and blocks the dopamine receptors at the same time. The mechanism of action of sigma receptors is not clear, but these can be involved in pain transition, anxiety, and modulation of muscle activity [38,39,40,41]. It is interesting to note that sigma receptors can also be involved in Parkinson’s disease [42]. In addition, these receptors were reported to exhibit anticonvulsant activity [43]. Taking into account the sigma receptors, previously studied amitriptyline is also a sigma-1 receptor agonist [44], but with a lower affinity than opipramol [45]. This may suggest that the efficacy of treatment with opipramol in the present study may be due to a higher global effect on sigma-1 receptor when compared to the Mohamed et al. study where the low doses of amitriptyline with low affinity to sigma-1 receptors were not reported effective [29]. Furthermore, the affinity of amitriptyline to sigma-2 receptors is not well known [42], while opipramol shows significant affinity to sigma-2 receptors [46]. It leads to the hypothesis that the specific role of sigma receptors in SB can be speculated upon.

Blocking the presynaptic D2 dopamine receptors leads to increased dopamine release, especially in the subcortical nuclei that regulate motor functions, including muscle tonus [47,48]. Studies have also indicated the correlation between dopaminergic mechanisms and SB [49,50]. In addition, a previous study suggested that rs686 within the dopamine receptor (DRD1) encoding gene may potentially affect predisposition to SB [51]. It is worth emphasizing that an activity similar to SB is one of the extrapyramidal symptoms of Parkinson’s disease and can be characterized by the reduced release of presynaptic dopamine [52,53]. The previously reported effects of dopaminergic agents on oral behaviors and the involvement of striatal D2 receptors in SB also support the potential role of the central dopaminergic system in bruxism [54,55]. Furthermore, Gomez et al. reported that haloperidol (D2, D3, D4 receptors inverse agonist and high affinity sigma-1 receptors antagonist) does not seem to modify bruxism on rat model [56]. Taking into account rather low presynaptic D2 receptor affinity of opipramol [20,21,22] and the fact that antipsychotics (in most cases D2 receptors antagonists) at standard clinical doses are reported to increase SB [54], it can be hypothesized that opipramol’s efficacy may be also related to presynaptic modulation.

It should be also emphasized that the fact that opipramol acts as a histamine H1 antagonist could also be considered significant in reducing SB. Histamine H1 receptors were reported to be potentially involved in masticatory muscles’ activity. Yoneda et al. in an experimental study reported that histamine H1 receptor antagonists may be effective in preventing prolonged masticatory muscles’ activity (also SB) [57]. There are also studies evaluating effect of blocking histamine H1 receptors on reducing SB in children. Ghanizadeh et al. reported that hydroxyzine intake decreased parent-reported SB in children [58]. Although, no instrumental methods of SB assessment were used in this study, this potentially relevant effect should also be further studied.

It should be also mentioned that bruxism is reported to be possibly associated with serotoninergic modulation [54]. Selective serotonin reuptake inhibitors (SSRI) in particular are reported to increase bruxism [59]. Desensitization of postsynaptic serotonin 2A (5HT_2A_) receptors mainly accompanies the intake of these drugs [60]. What is more, the serotonin 1A (5HT_1A_) receptors partial agonists such as buspirone have been reported to resolve the symptoms [59]. Opipramol acts as lower than SSRI affinity antagonist of 5HT_2A_ receptors [61], what can be also potentially contribute to its reported SB non-increasing effect.

Despite showing very promising but preliminary results confirming the effectiveness of opipramol in the management of severe SB, the present study has the following major limitations: it involved a small study group, as some participants did not attend the control vPSG examination; there was a lack of a control group and there was no non-active medication (placebo) use for second recording assessments; only a one-night vPSG examination was conducted because of the restrictions of the Polish health care system; there was a lack of possibility to perform an adaptive night; in a small group of patients the increase of BEI and BBI was observed. It should also be noted that, like any drug, opipramol may have long-term side effects and can be used only after excluding all of the contraindications. A longitudinal study is needed to clarify the possibility of developing side effects, and the maintenance of the observed results.

However, the preliminary results obtained seem to show potential clinical relevance. In the future, these observations may contribute to finding an effective drug that could be used in the management of severe SB.

## 5. Conclusions

A single 100 mg dose of opipramol at bedtime seems to positively affect reduction of SB in otherwise healthy individuals diagnosed with severe SB. Therefore the hypothesis that opipramol may be a possible drug in reducing severe SB could be accepted. However, the subject requires further research on a larger population including a control group. Further research should also focus on identifying the exact mechanism behind the opipramol-induced reduction of SB. Further studies should be focused on the clinical significance of SB parameters’ reduction and the meaning of this reduction for each type of muscular activity (phasic, tonic and mixed).

## Figures and Tables

**Table 1:** Descriptive statistics of stage I video polysomnography (vPSG) parameters (N = 19).

Parameter	Mean	Median	Minimum	Maximum	5th Percentile	95th Percentile	Standard Deviation
BEI (/h)	6.64	6.60	4.30	10.20	4,30	10.20	1.75
BBI (/h)	6.74	5.70	2.00	15.80	2.00	15.80	3.77
Apnea to bruxism (/h)	0.98	0.80	0.00	3.70	0.00	3.70	0.97
Arousal to bruxism (/h)	2.28	2.10	0.20	5.50	0.20	5.50	1.44
Phasic bruxism (/h)	4.23	4.50	0.10	8.00	0.10	8.00	2.12
Tonic bruxism (/h)	1.55	1.20	0.00	4.10	0.00	4.10	1.15
Mixed bruxism (/h)	0.96	0.90	0.10	1.90	0.10	1.90	0.54
AHI (/h)	2.45	2.20	0.50	6.60	0.50	6.60	1.63
ODI (/h)	2.58	2.10	0.10	6.90	0.10	6.90	1.90
Snore (%)	4.95	0.20	0.00	46.80	0.00	46.80	11.86
TST (m)	446.63	449.00	374.50	541.50	374.50	541.50	37.96
SL (m)	22.35	17.80	0.90	66.90	0.90	66.90	18.74
REML (m)	93.16	68.50	5.00	232.50	5.00	232.50	56.96
WASO (m)	23.18	15.50	0.00	84.30	0.00	84.30	22.39
SE (%)	88.72	90.30	73.60	99.00	73.60	99.00	6.89
N1 (%)	3.00	2.80	0.70	8.40	0.70	8.40	2.02
N2 (%)	45.16	47.20	23.30	61.30	23.30	61.30	10.07
N3 (%)	24.82	22.80	10.70	39.90	10.70	39.90	8.05
REM (%)	27.05	26.80	19.80	36.60	19.80	36.60	4.41
Arousals (/h)	4.77	4.10	0.40	12.60	0.40	12.60	3.55
Average SpO_2_ (%)	95.33	95.80	92.20	96.60	92.20	96.60	1.27
Minimal SpO_2_ (%)	88.47	91.00	64.00	96.00	64.00	96.00	7.51
SpO_2_ < 90% (%)	0.92	0.00	0.00	11.30	0.00	11.30	2.61
Average desaturation drop (%)	3.35	3.30	2.60	4.00	2.60	4.00	0.33
Average pulse (bpm)	61.41	58.90	50.90	78.10	50.90	78.10	8.26
Maximal pulse (bpm)	100.16	99.00	78.00	180.00	78.00	180.00	21.53
Minimal pulse (bpm)	49.42	47.00	38.00	68.00	38.00	68.00	7.81

BEI—bruxism episode index, BBI—bruxism burst index, AHI—apnea/hypopnea index, ODI—oxygen desaturation index, TST—total sleep time, SL—sleep latency, REML—rapid eye movement latency, WASO—wake after sleep onset, SE—sleep efficiency, N1—non-rapid eye movement 1, N2—non-rapid eye movement 2, N3—non-rapid eye movement 3, REM—rapid eye movement, /h—per hour of sleep, m—minutes, bpm—beats per minute.

**Table 2:** Descriptive statistics of stage II vPSG parameters (N = 19).

Parameter	Mean	Median	Minimum	Maximum	5th Percentile	95th Percentile	Standard Deviation
BEI	4.32	3.70	1.50	9.40	1.50	9.40	2.15
BBI	5.49	3.80	1.20	14.70	1.20	14.70	3.91
Apnea to bruxism	0.52	0.20	0.00	2.30	0.00	2.30	0.69
Arousal to bruxism	1.43	0.90	0.00	3.50	0.00	3.50	1.06
Phasic bruxism	2.54	2.10	0.00	7.70	0.00	7.70	1.97
Tonic bruxism	0.91	0.90	0.10	3.20	0.10	3.20	0.76
Mixed bruxism	0.79	0.70	0.00	3.80	0.00	3.80	0.78
AHI	2.36	2.00	0.00	7.40	0.00	7.40	2.06
ODI	3.14	2.00	0.00	8.00	0.00	8.00	2.44
Snore	5.64	0.20	0.00	52.10	0.00	52.10	14.84
TST	448.34	461.80	281.40	525.60	281.40	525.60	60.52
SL	22.81	11.60	0.40	130.50	0.40	130.50	30.85
REML	82.52	66.50	14.50	256.00	14.50	256.00	53.39
WASO	13.73	11.00	3.00	34.50	3.00	34.50	10.17
SE	89.76	93.40	58.40	98.40	58.40	98.40	10.28
N1	2.67	2.30	0.60	7.22	0.60	7.22	1.86
N2	46.95	48.10	27.60	70.50	27.60	70.50	10.80
N3	25.41	24.10	6.10	54.90	6.10	54.90	11.00
REM	24.97	25.70	15.30	34.30	15.30	34.30	5.81
Arousals	4.43	3.00	1.00	19.30	1.00	19.30	4.21
Average SpO_2_	95.15	95.50	93.20	96.70	93.20	96.70	0.96
Minimal SpO_2_	89.16	91.00	72.00	95.00	72.00	95.00	5.78
SpO_2_ < 90%	0.05	0.00	0.00	0.50	0.00	0.50	0.12
Average desaturation drop	3.03	3.10	0.00	3.80	0.00	3.80	0.77
Average pulse	61.93	59.30	48.90	75.70	71.00	155.00	8.82
Maximal pulse	94.89	93.00	71.00	155.00	48.90	75.70	17.11
Minimal pulse	49.84	47.00	38.00	64.00	38.00	64.00	7.68

BEI—bruxism episode index, BBI—bruxism burst index, AHI—apnea/hypopnea index, ODI—oxygen desaturation index, TST—total sleep time, SL—sleep latency, REML—rapid eye movement latency, WASO—wake after sleep onset, SE—sleep efficiency, N1—non-rapid eye movement 1, N2—non-rapid eye movement 2, N3—non-rapid eye movement 3, REM—rapid eye movement.

**Table 3:** Change in the studied parameters after pharmacotherapy (N = 19).

Parameter	Mean	Median	Minimum	Maximum	5th Percentile	95th Percentile	Standard Deviation	*p*
BEI change	−2.33	−2.80	−5.80	2.90	−5.80	2.90	2.41	0.00
BBI change	−1.25	−1.80	−12.50	8.20	−12.50	8.20	4.88	0.28
Apnea to bruxism change	–0.46	–0.20	–3.70	0.70	−3.70	0.70	0.99	0.06
Arousal to bruxism change	–0.85	–0.50	–4.60	3.00	−4.60	3.00	1.72	0.04
Phasic bruxism change	–1.68	–2.20	–3.70	3.20	−3.70	3.20	1.79	0.00
Tonic bruxism change	–0.64	–0.60	–2.70	1.30	−2.70	1.30	1.15	0.03
Mixed bruxism change	–0.17	–0.20	–1.40	2.30	−1.40	2.30	0.87	0.41
AHI change	–0.09	–0.30	–3.60	4.60	−3.60	4.60	1.71	0.82
ODI change	0.56	0.80	–4.40	6.40	−4.40	6.40	2.18	0.28
Snore change	0.69	0.00	–10.90	26.80	−10.90	26.80	6.99	0.67
TST change	1.71	11.90	–196.90	136.90	−196.90	136.90	77.64	0.92
SL change	0.46	–0.80	–66.50	81.10	−66.50	81.10	29.63	0.95
REML change	–10.64	–2.50	–152.00	108.50	−152.00	108.50	59.66	0.45
WASO change	–9.46	–8.50	–59.00	22.50	−59.00	22.50	20.84	0.06
SE change	1.04	3.10	–28.50	23.40	−28.50	23.40	10.26	0.66
N1 change	–0.33	–0.50	–3.80	4.90	−3.80	4.90	2.52	0.58
N2 change	1.79	0.20	–10.90	12.70	−10.90	12.70	6.90	0.27
N3 change	0.59	0.90	–16.10	20.90	−16.10	20.9	8.11	0.76
REM change	–2.07	–0.40	–12.10	8.00	−12.10	8.00	5.33	0.11
Arousals change	–0.35	–0.10	–9.70	14.20	−9.70	14.20	4.97	0.76
Average SpO_2_ change	–0.18	–0.50	−2.00	2.20	−2.00	2.20	1.10	0.49
Minimal SpO_2_ change	0.68	1.00	–17.00	12.00	−17.00	12.00	7.09	0.68
SpO_2_ < 90% change	–0.86	0.00	–11.30	0.50	−11.30	0.50	2.63	0.17
Average desaturation drop change	–0.32	–0.30	−3.40	0.70	−3.40	0.70	0.85	0.12
Average pulse	0.52	−1.10	−3.40	15.20	−3.40	15.20	5.20	0.67
Maximal pulse	−5.26	−3.00	−28.00	8.00	−28.00	8.00	9.16	0.02
Minimal pulse	0.42	0.00	−6.00	14.00	−6.00	14.00	5.24	0.73

Student’s *t*-test was used for normally distributed variables. In the case of variables with a distribution other than normal, the Mann–Whitney U-test was used. BEI—bruxism episode index, BBI—bruxism burst index, AHI—apnea/hypopnea index, ODI—oxygen desaturation index, TST—total sleep time, SL—sleep latency, REML—rapid eye movement latency, WASO—wake after sleep onset, SE—sleep efficiency, N1—non-rapid eye movement 1, N2—non-rapid eye movement 2, N3—non-rapid eye movement 3, REM—rapid eye movement.

**Table 4:** Comparison of change in studied parameters between group 1 (SB improvement) and group 3 (SB aggravation).

Parameter	Group 1				Group 3				*p*
	Mean	5th Percentile	95th Percentile	SD	Mean	5th Percentile	95th Percentile	SD	
BEI change	−3.35	−5.80	−1.60	1.29	2.80	2.70	2.90	0.14	0.00
BBI change	−2.84	−12.50	1.60	3.68	7.50	6.80	8.20	0.99	0.00
Apnea to bruxism change	−0.50	−3.70	0.70	1.04	−0.05	−0.20	0.10	0.21	0.56
Arousal to bruxism change	−0.92	−4.60	3.00	1.87	0.00	−0.50	0.50	0.71	0.51
Phasic bruxism change	−2.25	−3.70	0.50	0.97	1.15	−0.90	3.20	2.90	0.00
Tonic bruxism change	−0.76	−2.70	1.30	1.07	−0.10	−1.40	1.20	1.84	0.45
Mixed bruxism change	−0.35	−1.40	1.10	0.69	1.15	0.00	2.30	1.63	0.02
AHI change	0.12	−3.60	4.60	1.88	−0.95	−1.00	−0.90	0.07	0.45
ODI change	0.51	−4.40	6.40	2.46	0.90	0.80	1.00	0.14	0.83
Snore change	0.71	−10.90	26.80	7.90	−0.20	−0.20	−0.20	0.00	0.88
TST change	15.33	−111.90	136.90	63.02	−88.85	−196.90	19.20	152.81	0.08
SL change	7.09	−25.40	81.10	27.02	−39.15	−66.50	−11.80	38.68	0.04
REML change	−5.63	−152.00	108.50	64.84	−18.80	−25.10	−12.50	8.91	0.78
WASO change	−14.00	−59.00	15.00	20.18	5.50	−11.50	22.50	24.04	0.22
SE change	2.67	−11.10	23.40	8.26	−11.85	−28.50	4.80	23.55	0.07
N1 change	−0.72	−3.80	3.72	2.46	2.20	−0.50	4.90	3.82	0.15
N2 change	1.71	−10.90	12.70	7.15	7.05	2.80	11.30	6.01	0.33
N3 change	−0.13	−16.10	20.90	8.52	−0.40	−3.00	2.20	3.68	0.97
REM change	−0.87	−12.10	8.00	5.04	−8.90	−9.90	−7.90	1.41	0.05
Arousals change	−0.37	−9.70	14.20	5.55	1.30	0.30	2.30	1.41	0.69
Average SpO_2_ change	0.05	−1.20	2.20	1.08	−1.25	−2.00	−0.50	1.06	0.13
Minimal SpO_2_ change	1.40	−17.00	12.00	7.81	−1.00	−2.00	0.00	1.41	0.68
SpO_2_ < 90% change	−1.13	−11.30	0.10	2.92	0.00	0.00	0.00	0.00	0.60
Average desaturation drop change	−0.48	−3.40	0.10	0.87	0.10	−0.30	0.50	0.57	0.38
Average pulse change	1.03	−3.40	15.20	5.74	−0.40	−1.70	0.90	1.84	0.74
Maximal pulse change	−5.33	−28.00	8.00	10.02	−2.50	−7.00	2.00	6.36	0.71
Minimal pulse change	0.73	−6.00	14.00	5.74	1.50	0.00	3.00	2.12	0.86

Student’s *t*-test was used for normally distributed variables. In the case of variables with a distribution other than normal, the Mann–Whitney U-test was used. SD—standard deviation, BEI—bruxism episode index, BBI—bruxism burst index, AHI—apnea/hypopnea index, ODI—oxygen desaturation index, TST—total sleep time, SL—sleep latency, REML—rapid eye movement latency, WASO—wake after sleep onset, SE—sleep efficiency, N1—non-rapid eye movement 1, N2—non-rapid eye movement 2, N3—non-rapid eye movement 3, REM—rapid eye movement.

**Table 5:** Comparison of the change in the studied parameters between genders.

Parameter	Female (N = 14)				Male (N = 5)				*p*
	Mean	5th Percentile	95th Percentile	SD	Mean	5th Percentile	95th Percentile	SD	
BEI change	−2.36	−5.80	2.70	2.27	−2.24	−5.00	2.90	3.05	0.93
BBI change	−2.21	−12.50	6.80	4.93	1.42	−2.00	8.20	4.01	0.16
Apnea to bruxism change	−0.58	−3.70	0.70	1.10	−0.14	−0.60	0.70	0.55	0.41
Arousal to bruxism change	−1.11	−4.60	3.00	1.92	−0.14	−1.10	0.50	0.67	0.29
Phasic bruxism change	−1.76	−3.50	2.10	1.48	−1.46	−3.70	3.20	2.68	0.75
Tonic bruxism change	−0.44	−2.70	1.30	1.27	−1.20	−1.90	−0.60	0.49	0.22
Mixed bruxism change	−0.28	−1.40	2.30	0.93	0.14	−0.60	1.10	0.67	0.37
AHI change	−0.61	−3.60	2.40	1.30	1.38	−0.90	4.60	2.00	0.02
ODI change	−0.04	−4.40	2.70	1.84	2.24	1.00	6.40	2.34	0.04
Snore change	1.46	−10.90	26.80	7.97	−1.44	−4.10	1.10	2.43	0.44
TST change	−11.39	−196.90	92.00	78.67	38.40	−35.20	136.90	68.75	0.23
SL change	4.42	−66.50	81.20	32.27	−10.64	−25.40	22.00	18.89	0.34
REML change	−16.19	−152.00	108.50	68.24	4.90	−16.00	32.50	21.70	0.51
WASO change	−9.91	−59.00	22.50	23.60	−8.20	−25.50	6.50	11.96	0.88
SE change	−0.67	−28.50	8.40	9.89	5.82	−4.40	23.40	10.79	0.23
N1 change	−0.56	−3.80	4.90	2.83	0.32	−1.10	2.10	1.36	0.52
N2 change	0.56	−10.90	9.90	6.33	5.22	−4.90	12.70	8.02	0.20
N3 change	2.84	−6.20	20.90	7.32	−5.72	−16.10	1.60	7.38	0.04
REM change	−2.86	−12.20	8.00	5.44	0.12	−7.90	4.30	4.87	0.30
Arousals change	−0.79	−9.70	14.20	5.76	0.90	0.10	2.20	0.86	0.53
Average SpO_2_ change	−0.16	−2.00	2.20	1.19	−0.24	−1.20	1.00	0.88	0.89
Minimal SpO_2_ change	0.64	−17.00	12.00	7.49	0.80	−7.00	11.00	6.61	0.97
SpO_2_ < 90% change	−0.92	−11.30	0.50	3.02	−0.70	−2.90	0.00	1.26	0.88
Average desaturation drop change	−0.41	−3.40	0.70	0.98	−0.06	−0.30	0.10	0.17	0.44
Average pulse change	0.98	−3.40	15.20	5.98	−0.76	−2.10	1.10	1.61	0.54
Maximal pulse change	−6.71	−28.00	8.00	10.16	−1.20	−7.00	2.00	3.83	0.26
Minimal pulse change	0.50	−6.00	14.00	5.97	0.20	−3.00	3.00	2.77	0.92

Student’s *t*-test was used for normally distributed variables. In the case of variables with a distribution other than normal, the Mann–Whitney U-test was used. SD—standard deviation, BEI—bruxism episode index, BBI—bruxism burst index, AHI—apnea/hypopnea index, ODI—oxygen desaturation index, TST—total sleep time, SL—sleep latency, REML—rapid eye movement latency, WASO—wake after sleep onset, SE—sleep efficiency, N1—non-rapid eye movement 1, N2—non-rapid eye movement 2, N3—non-rapid eye movement 3, REM—rapid eye movement.

## Data Availability

The data presented in this study are available on request from the corresponding author. The data are not publicly available due to European Union restrictions.

## References

[1] Lobbezoo F., Ahlberg J., Raphael K.G., Wetselaar P., Glaros A.G., Kato T., Santiago V., Winocur E., De Laat A., De Leeuw R. (2018). International consensus on the assessment of bruxism: Report of a work in progress. J. Oral. Rehabil..

[2] Raphael K.G., Santiago V., Lobbezoo F. (2016). Is bruxism a disorder or a behavior? Rethinking the international consensus on defining and grading of bruxism. J. Oral. Rehabil..

[3] Manfredini D., Serra-Negra J., Carboncini F., Lobbezoo F. (2017). Current concepts of bruxism. Int. J. Prosthodont..

[4] Lobbezoo F., Ahlberg J., Glaros A.G., Kato T., Koyano K., Lavigne G.J., de Leeuw R., Manfredini D., Svensson P., Winocur E. (2013). Bruxism defined and graded: An international consensus. J. Oral. Rehabil..

[5] American Academy of Sleep Medicine (2014). International Classification of Sleep Disorders.

[6] Beddis H., Pemberton M., Davies S. (2018). Sleep bruxism: An overview for clinicians. Br. Dent. J..

[7] Jokubauskas L., Baltrušaitytė A., Pileičikienė G. (2018). Oral appliances for managing sleep bruxism in adults: A systematic review from 2007 to 2017. J. Oral. Rehabil..

[8] Luiz de Barreto Aranha R., Nogueira Guimarães de Abreu M.H., Serra-Negra J.M., Martins R.C. (2018). Evidence-based support for sleep bruxism treatment other than oral appliances remains insufficient. J. Evid. Based. Dent. Pract..

[9] Singh P.K., Alvi H.A., Singh B.P., Singh R.D., Kant S., Jurel S., Singh K., Arya D., Dubey A. (2015). Evaluation of various treatment modalities in sleep bruxism. J. Prosthet. Dent..

[10] Amorim C.S.M., Espirito Santo A.S., Sommer M., Marques A.P. (2018). Effect of physical therapy in bruxism treatment: A systematic review. J. Manip. Physiol. Ther..

[11] Santos Miotto Amorim C., Firsoff E.F., Vieira G.F., Costa J.R., Marques A.P. (2014). Effectiveness of two physical therapy interventions, relative to dental treatment in individuals with bruxism: Study protocol of a randomized clinical trial. Trials.

[12] Kijak E., Lietz-Kijak D., Sliwiński Z., Frączak B. (2013). Muscle activity in the course of rehabilitation of masticatory motor system functional disorders. Postepy Hig. Med. Dosw..

[13] Mesko M.E., Hutton B., Skupien J.A., Sarkis-Onofre R., Moher D., Pereira-Cenci T. (2017). Therapies for bruxism: A systematic review and network meta-analysis (protocol). Syst. Rev..

[14] Valiente López M., van Selms M.K., van der Zaag J., Hamburger H.L., Lobbezoo F. (2015). Do sleep hygiene measures and progressive muscle relaxation influence sleep bruxism? Report of a randomised controlled trial. J. Oral. Rehabil..

[15] Tinastepe N., Küçük B.B., Oral K. (2015). Botulinum toxin for the treatment of bruxism. Cranio.

[16] Kumar A., Spivakovsky S. (2018). Bruxism- is botulinum toxin an effective treatment?. Evid. Based. Dent..

[17] Macedo C.R., Macedo E.C., Torloni M.R., Silva A.B., Prado G.F. (2014). Pharmacotherapy for sleep bruxism. Cochrane Database Syst. Rev..

[18] Winocur E., Gavish A., Voikovitch M., Emodi-Perlman A., Eli I. (2003). Drugs and bruxism: A critical review. J. Orofac. Pain..

[19] Cahlin B.J., Hedner J., Dahlström L. (2017). A randomised, open-label, crossover study of the dopamine agonist, pramipexole, in patients with sleep bruxism. J. Sleep Res..

[20] Gahr M., Hiemke C., Connemann B.J. (2017). Update opipramol [update opipramol]. Fortschr. Neurol. Psychiatr..

[21] Krysta K., Murawiec S., Warchala A., Zawada K., Cubała W.J., Wiglusz M.S., Jakuszkowiak-Wojten K., Krzystanek M., Krupka-Matuszczyk I. (2015). Modern indications for the use of opipramol. Psychiatr. Danub..

[22] Fun H.K., Loh W.S., Siddegowda M.S., Yathirajan H.S., Narayana B. (2011). Opipramol. Acta Crystallogr. Sect. E Struct. Rep..

[23] Gillman P.K. (2007). Tricyclic antidepressant pharmacology and therapeutic drug interactions updated. Br. J. Pharmacol..

[24] Schneider J., Patterson M., Jimenez X.F. (2019). Beyond depression: Other uses for tricyclic antidepressants. Cleve Clin. J. Med..

[25] Gerlach K., Uhlig T., Plathof J., Klassen A., Stoll K.D., Schmucker P., Hueppe M. (2002). Effects of opipramol as an evening anaesthesiologic premedication. Neuropsychobiology.

[26] Hueppe M., Hartge D., Stoll K.D., Ros A., Schmucker P., Gerlach K. (2011). Opipramol improves subjective quality of sleep the night prior to surgery: Confirmatory testing of a double-blind, randomized clinical trial. Neuropsychobiology.

[27] Berry R.B., Budhiraja R., Gottlieb D.J., Gozal D., Iber C., Kapur V.K., Marcus C.L., Mehra R., Parthasarathy S., Quan S.F. (2012). American Academy of Sleep Medicine. Rules for scoring respiratory events in sleep: Update of the 2007 AASM manual for the scoring of sleep and associated events. Deliberations of the sleep apnea definitions task force of the American academy of sleep medicine. J. Clin. Sleep Med..

[28] Mohamed S.E., Christensen L.V., Penchas J. (1997). A randomized double-blind clinical trial of the effect of amitriptyline on nocturnal masseteric motor activity (sleep bruxism). Cranio.

[29] Raigrodski A.J., Mohamed S.E., Gardiner D.M. (2001). The effect of amitriptyline on pain intensity and perception of stress in bruxers. J. Prosthodont..

[30] Raigrodski A.J., Christensen L.V., Mohamed S.E., Gardiner D.M. (2001). The effect of four-week administration of amitriptyline on sleep bruxism. A double-blind crossover clinical study. Cranio.

[31] Şahin Onat S., Malas F.Ü. (2015). Duloxetine-induced sleep bruxism in fibromyalgia successfully treated with amitriptyline. Acta Reumatol. Port..

[32] Huynh N., Lavigne G.J., Lanfranchi P.A., Montplaisir J.Y., de Champlain J. (2006). The effect of 2 sympatholytic medications--propranolol and clonidine—On sleep bruxism: Experimental randomized controlled studies. Sleep.

[33] Lavigne G.J., Soucy J.P., Lobbezoo F., Manzini C., Blanchet P.J., Montplaisir J.Y. (2001). Double-blind, crossover, placebo-controlled trial of bromocriptine in patients with sleep bruxism. Clin. Neuropharmacol..

[34] Huynh N., Guitard F., Manzine C., Montplaisir J.Y., De Champlain J., Lavigne G.J. (2004). Lack of effect of propranolol on sleep bruxism: A controlled double-blind study. Sleep.

[35] Etzel K.R., Stockstill J.W., Rugh J.D., Fisher J.G. (1991). Tryptophan supplementation for nocturnal bruxism: Report of negative results. J. Craniomandib. Disord..

[36] Lobbezoo F., Lavigne G.J., Tanguay R., Montplaisir J.Y. (1996). Does L-dopa exacerbate sleep bruxism?. J. Oral. Rehabil..

[37] Lobbezoo F., Lavigne G.J., Tanguay R., Montplaisir J.Y. (1997). Theeffect of catecholamine precursor L-dopa on sleep bruxism: A controlled clinical trial. Mov. Disord..

[38] Furuse T., Hashimoto K. (2009). Fluvoxamine monotherapy for psychotic depression: The potential role of sigma-1 receptors. Ann. Gen. Psychiatry.

[39] Stahl S.M. (2005). Antidepressant treatment of psychotic major depression: Potential role of the sigma receptor. CNS Spectr..

[40] Campbell B.G., Scherz M.W., Keana J.F., Weber E. (1989). Sigma receptors regulate contractions of the guinea pig ileum longitudinal muscle/myenteric plexus preparation elicited by both electrical stimulation and exogenous serotonin. J. Neurosci..

[41] Mancuso R., Navarro X. (2017). Sigma-1 Receptor in Motoneuron Disease. Adv. Exp. Med. Biol..

[42] Yang K., Wang C., Sun T. (2019). The roles of intracellular chaperone proteins, sigma receptors, in Parkinson’s Disease (PD) and Major Depressive Disorder (MDD). Front. Pharmacol..

[43] Keshavarz M., Yekzaman B. (2018). Amelioration of pentylenetetrazole-induced seizures by modulators of sigma, N-Methyl-D-Aspartate, and ryanodine receptors in mice. Iran. J. Med. Sci..

[44] Weber E., Sonders M., Quarum M., McLean S., Pou S., Keana J.F. (1986). 1,3-Di(2-[5-3H]tolyl)guanidine: A selective ligand that labels sigma-type receptors for psychotomimetic opiates and antipsychotic drugs. Proc. Natl. Acad. Sci. USA.

[45] Hanner M., Moebius F.F., Flandorfer A., Knaus H.G., Striessnig J., Kempner E., Glossmann H. (1996). Purification, molecular cloning, and expression of the mammalian sigma1-binding site. Proc. Natl. Acad. Sci. USA.

[46] Sills M.A., Loo P.S. (1989). Tricyclic antidepressants and dextromethorphan bind with higher affinity to the phencyclidine receptor in the absence of magnesium and L-glutamate. Mol. Pharmacol..

[47] Thorstensen J.R., Tucker M.G., Kavanagh J.J. (2018). Antagonism of the D2 dopamine receptor enhances tremor but reduces voluntary muscle activation in humans. Neuropharmacology.

[48] Lanciego J.L., Luquin N., Obeso J.A. (2012). Functional neuroanatomy of the basal ganglia. Cold Spring Harb. Perspect. Med..

[49] Laat A., Macaluso G.M. (2020). Sleep bruxism as a motor disorder. Mov. Disord..

[50] Lobbezoo F., Naeije M. (2001). Bruxism is mainly regulated centrally, not peripherally. J. Oral. Rehabil..

[51] Wieckiewicz M., Bogunia-Kubik K., Mazur G., Danel D., Smardz J., Wojakowska A., Poreba R., Dratwa M., Chaszczewska-Markowska M., Winocur E. (2020). Genetic basis of sleep bruxism and sleep apnea-response to a medical puzzle. Sci. Rep..

[52] McGuigan S., Zhou S.H., Brosnan M.B., Thyagarajan D., Bellgrove M.A., Chong T.T. (2019). Dopamine restores cognitive motivation in Parkinson′s disease. Brain.

[53] Zlotnik Y., Balash Y., Korczyn A.D., Giladi N., Gurevich T. (2015). Disorders of the oral cavity in Parkinson’s disease and parkinsonian syndromes. Parkinsons. Dis..

[54] Falisi G., Rastelli C., Panti F., Maglione H., Quezada Arcega R. (2014). Psychotropic drugs and bruxism. Expert. Opin. Drug Saf..

[55] Lobbezoo F., Soucy J.P., Montplaisir J.Y., Lavigne G.J. (1996). Striatal D2 receptor binding in sleep bruxism: A controlled study with iodine-123-iodobenzamide and single-photon-emission computed tomography. J. Dent. Res..

[56] Gómez F.M., Areso M.P., Giralt M.T., Sainz B., García-Vallejo P. (1998). Effects of dopaminergic drugs, occlusal disharmonies, and chronic stress on non-functional masticatory activity in the rat, assessed by incisal attrition. J. Dent. Res..

[57] Yoneda H., Niijima-Yaoita F., Tsuchiya M., Kumamoto H., Watanbe M., Ohtsu H., Yanai K., Tadano T., Sasaki K., Sugawara S. (2013). Roles played by histamine in strenuous or prolonged masseter muscle activity in mice. Clin. Exp. Pharmacol. Physiol..

[58] Ghanizadeh A., Zare S. (2013). A preliminary randomised double-blind placebo-controlled clinical trial of hydroxyzine for treating sleep bruxism in children. J. Oral. Rehabil..

[59] Garrett A.R., Hawley J.S. (2018). SSRI-associated bruxism: A systematic review of published case reports. Neurol. Clin. Pract..

[60] Celada P., Puig M., Amargós-Bosch M., Adell A., Artigas F. (2004). The therapeutic role of 5-HT1A and 5-HT2A receptors in depression. J. Psychiatry Neurosci..

[61] Holoubek G., Müller W.E. (2003). Specific modulation of sigma binding sites by the anxiolytic drug opipramol. J. Neural. Transm..

